# Prediction of m5C Modifications in RNA Sequences by Combining Multiple Sequence Features

**DOI:** 10.1016/j.omtn.2020.06.004

**Published:** 2020-06-10

**Authors:** Lijun Dou, Xiaoling Li, Hui Ding, Lei Xu, Huaikun Xiang

**Affiliations:** 1 School of Automotive and Transportation Engineering, Shenzhen Polytechnic, Shenzhen, China; 2 Institute of Fundamental and Frontier Sciences, University of Electronic Science and Technology of China, Chengdu, China; 3 Department of Oncology, Heilongjiang Province Land Reclamation Headquarters General Hospital, Harbin, China; 4 Center for Informational Biology, University of Electronic Science and Technology of China, Chengdu, China; 5 School of Electronic and Communication Engineering, Shenzhen Polytechnic, Shenzhen, China

**Keywords:** 5-methylcytosine, position-specific propensity, nucleotide composition, electron-ion interaction pseudopotentials of trinucleotide, PC-PseDNC-general, support vector machine

## Abstract

5-Methylcytosine (m5C) is a well-known post-transcriptional modification that plays significant roles in biological processes, such as RNA metabolism, tRNA recognition, and stress responses. Traditional high-throughput techniques on identification of m5C sites are usually time consuming and expensive. In addition, the number of RNA sequences shows explosive growth in the post-genomic era. Thus, machine-learning-based methods are urgently requested to quickly predict RNA m5C modifications with high accuracy. Here, we propose a noval support-vector-machine (SVM)-based tool, called iRNA-m5C_SVM, by combining multiple sequence features to identify m5C sites in *Arabidopsis thaliana*. Eight kinds of popular feature-extraction methods were first investigated systematically. Then, four well-performing features were incorporated to construct a comprehensive model, including position-specific propensity (PSP) (PSNP, PSDP, and PSTP, associated with frequencies of nucleotides, dinucleotides, and trinucleotides, respectively), nucleotide composition (nucleic acid, di-nucleotide, and tri-nucleotide compositions; NAC, DNC, and TNC, respectively), electron-ion interaction pseudopotentials of trinucleotide (PseEIIPs), and general parallel correlation pseudo-dinucleotide composition (PC-PseDNC-general). Evaluated accuracies over 10-fold cross-validation and independent tests achieved 73.06% and 80.15%, respectively, which showed the best predictive performances in *A. thaliana* among existing models. It is believed that the proposed model in this work can be a promising alternative for further research on m5C modification sites in plant.

## Graphical Abstract

**Figure:**
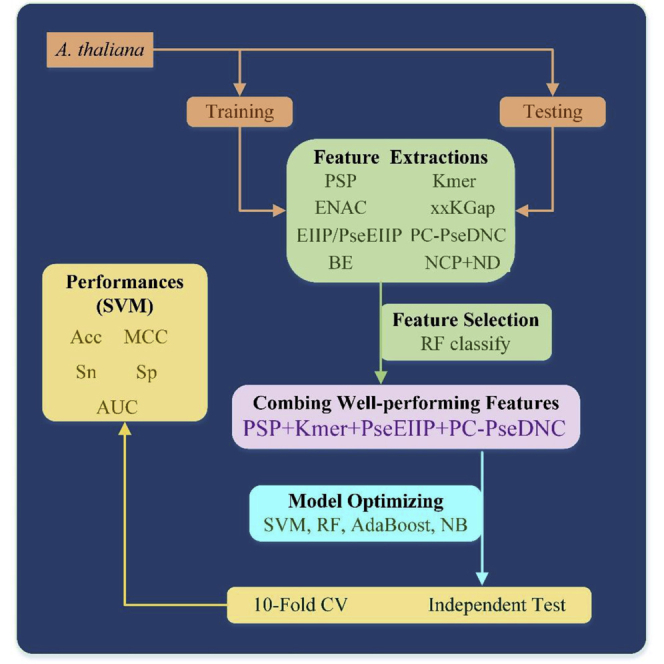

## Teaser

5-Methylcytosine (m5C) is a well-known post-transcriptional modification, which plays a significant role in various biological processes. Dou et al. built a novel SVM-based predictor, called iRNA-m5C_SVM, to identify RNA m5C modifications using multiple sequence features. Corresponding performances were performed with other reported methods, which provided a competitive bioinformatic tool to predict m5C sites.

## Introduction

To date, more than 150 types of RNA post-transcriptional modifications have been found in all kingdoms of life.^1^, ^2^, ^3^, ^4^, ^5^, ^6^, ^7^ As one of most prevalent modifications, 5-methylcytosine (m5C) is catalyzed by RNA methyltransferase, in which a methyl group is attached to the fifth position of the cytosine ring. It has been reported that m5C sites are involved in many kinds of biological processes, including RNA structural stability and metabolism, tRNA recognition and stress responses,^8^, ^9^, ^10^, ^11^, ^12^, ^13^, ^14^ and so forth. Additionally, it has also been proved that m5c modifications are associated with many diseases, such as breast cancer,^15^ autosomal recessive intellectual disability,^16^ amyotrophic lateral sclerosis,^17^ and Parkinson’s disease.^18^ Thus, the accurate identification of m5C is the primary and crucial task for carrying out the research on corresponding diseases and biological functions.^8^^,^^9^^,^^11^, ^12^, ^13^^,^^15^, ^16^, ^17^, ^18^, ^19^, ^20^, ^21^ In experiments, several traditional high-throughput sequencing techniques, such as bisulfite conversion,^22^ miCLIP,^23^ and Aza-IP,^24^ have been developed to detect m5C sites. More details about m5C biological mechanisms and related diseases can be found in Chen et al.^25^ and literature therein. However, considering the time-consuming and labor-intensive nature of these techniques, it is challenging to keep pace with the dramatic increase of the number of RNA sequences in the post-genome era. Therefore, the identification of m5C and non-m5C sequences using computational methods is of great significance and necessity.

Eight computational predictors have been proposed to detect m5C sites in RNA sequences, including m5C-PseDNC,^26^ iRNAm5C-PseDNC,^27^ M5C-HPCR,^28^ pM^5^CS-Comp-mRMR,^29^ RNAm5Cfinder,^30^ PEA-m5C,^31^ iRNA-m5C,^32^ and RNAm5CPred.^33^ Related species, feature-extraction techniques, and classifiers are listed in Table 1. It can be seen that there were a total of four species investigated: *Homo sapiens*, *Mus musculus*, *Saccharomyces cerevisiae*, and *Arabidopsis thaliana*. In specific, Feng et al.^26^ first provided the m5C-PseDNC tool based on the support vector machine (SVM) in *H. sapiens*. By applying pseudo-dinucleotide composition (PseDNC) features with three physiochemical properties, the accuracy over the jackknife test achieved 90.42%. Qiu et al.^27^ also used PseDNC features with 10 properties to construct the random forest (RF) model called iRNAm5C-PseDNC, where the jackknife test gave an accuracy of 92.37%. Later, Zhang et al.^28^ introduced the m5c-HPCR model, with a higher Matthew’s correlation coefficient (MCC) of 0.859 and area under the receiver operating characteristic (ROC) curve (AUC) of 0.962, where a novel heuristic nucleotide physicochemical property reduction (HPCR) algorithm was applied. Then, Sabooh et al.^29^ presented the pM^5^CS-Comp-mRMR method, with an accuracy of 93.33%, where the minimum redundancy and maximum relevance (mRMR) method was used to select effective features from Kmer features with *k*s = 2, 3, and 4 (corresponding to di-nucleotide composition, tri-nucleotide composition, and tetra-nucleotide composition; DNC, TNC, and TetraNC, respectively). For the m5C sites in *A. thaliana*, Song et al.^31^ first developed the predictor PEA-M5C, where an independent test showed an overall accuracy of 83.5% with the MCC of 0.688. In this method, three kinds of feature-encoding techniques—binary encoding (BE), Kmer, and PseDNC—were incorporated to give combined performances. Li et al.^30^ designed the RNAm5Cfinder using BE features to analyze m5C sites in *H. sapiens* and *M. musculus*, where comprehensive and cell-specific predictors gave AUC values of 0.77 and 0.87, respectively. Recently, Lv et al.^32^ established a novel approach, iRNA-m5C, to systematically diagnose m5C sites in four species, where Kmer, BE, pseudo-k-tuple nucleotide composition (PseKNC), and natural vector (NV) were incorporated to obtain overall results. Optimal models of four species gave evaluated accuracies of 92.90%, 100.00%, 100.00%, and 70.70% on training datasets and 74.00% on testing datasets in *A. thaliana*. Also recently, Fang et al.^33^ constructed an accurate RNAm5CPred tool in *H. sapiens*, where Kmer (described as K-nucleotide frequencies [KNFs] in their paper), K-spaced nucleotide pair frequencies (KSNPFs), and PseDNC were combined to represent RNA samples.

**Table 1:** Eight Proposed Methods to Identify m5C Sites in RNA Sequences

Method	Species	Feature Extraction/Selection	Classifiers
m5C-PseDNC^26^	*H. sapiens*	PseDNC (3 properties)	SVM
iRNAm5C-PseDNC^27^	*H. sapiens*	PseDNC (10 properties)	RF
M5C-HPCR^28^	*H. sapiens*	HPCR	SVM
pM^5^CS-Comp-mRMR^29^	*H. sapiens*	Kmer (k = 2, 3, and 4) /mRMR	SVM
RNAm5Cfinder^30^	*H. sapiens*, *M. musculus*	BE	RF
PEA-m5C^31^	*A. thaliana*	BE + Kmer + PseDNC	RF
iRNA-m5C^32^	*H. sapiens*, *S. cerevisiae*, *M. musculus*, *A. thaliana*	Kmer + BE + NV + PseKNC	RF
RNAm5CPred^33^	*H. sapiens*	Kmer + KSNPF + PseDNC	SVM

Generally, except for the PEA-M5C^31^ model, which was focused on *A. thaliana*, seven other tools^26^, ^27^, ^28^, ^29^, ^30^^,^^32^^,^^33^ all gave better performances in *H. sapiens*, where the average accuracy was higher than 90%. As for *S. cerevisiae* and *M. musculus*, it was noted that only 97 and 211 positive samples were experimentally validated, where the remaining sequences, by removing sequence similarity, were too few to construct computational predictors (i.e., lacking of statistical significance; details can be found in Sun et al.^5^ and Lv et al.^32^). In addition, reported accuracies using the original data were adequately equal to 100.00%. It is hoped that more ideal/reliable models will be built in the future, with more experiment-proven sequences. As for the only plant, *A. thaliana*, there were only two predictors developed: PEA-m5C^31^ and iRNA-m5C.^32^ Especially, the latest iRNAm5C method presented accuracies of 70.7% and 74% over 10-fold cross-validation (CV) and independent tests using combined features “KNFC + MNBE + NV,” respectively. On the other hand, only a few feature-extraction techniques have been used in two published methods. Therefore, there is still a big hope for improving predictive performances by applying other new feature-encoding techniques. In summary, we were mainly focused on improving the performances of the identification of m5C sites in *A. thaliana* in this article (Table 1).

**Figure 1:**
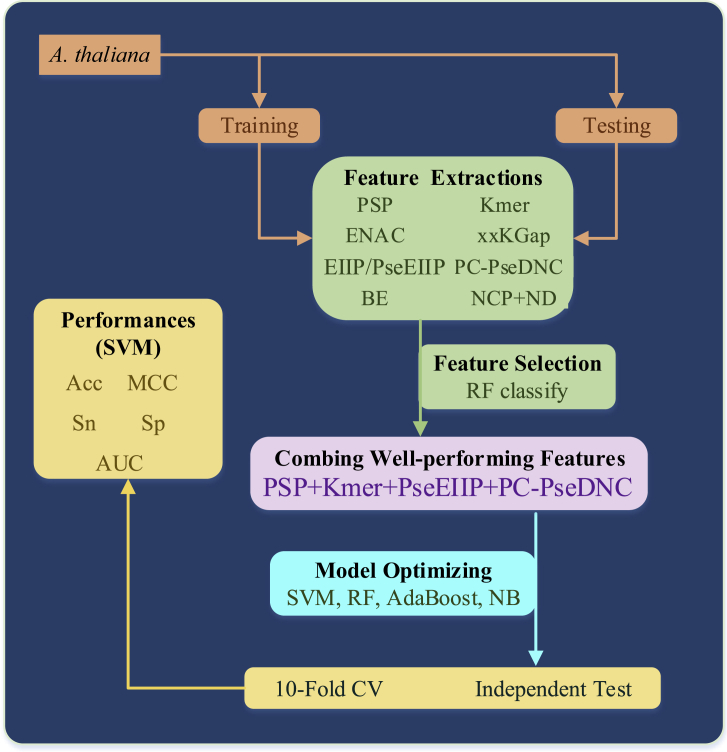
The Flowchart of the Proposed Predictor for m5C Identification by Combining Multiple Sequence Features

We first investigated eight kinds of sequence-representing methods; namely, position-specific propensity (PSP), Kmer, enhanced nucleic acid composition (ENAC), xxKGap, electron-ion interaction pseudopotentials (EIIPs) and EIIPs of trinucleotides (PseEIIPs), general parallel correlation PseDNC (PC-PseDNC-general), nucleotide chemical property and nucleotide density (NCP + ND), and BE. Then, four well-performing features, “PSP + Kmer + PseEIIP + PseDNC,” chosen by preliminary results, were incorporated to build the prediction model. Four different classifiers (SVM, RF, AdaBoost, and Naive Bayes [NB]) were separately applied for comparison, where the best performing model was optimized using the SVM method. The schematic flowchart of this work is shown in Figure 1.

## Results and Discussion

### Predictive Performances Using One Kind of Feature

**Figure 2:**
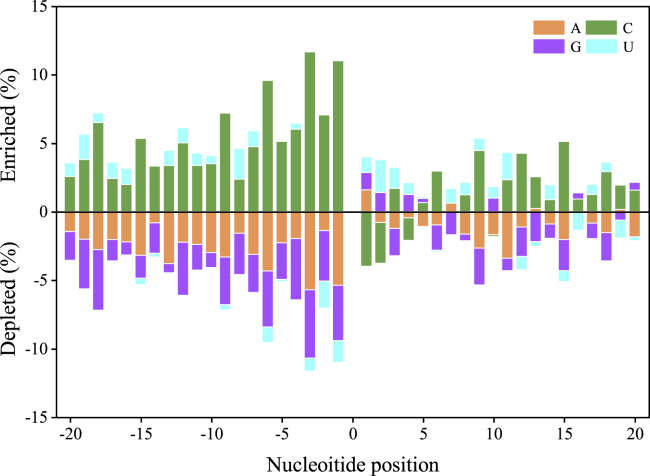
Differences of Position-Specific Nucleotide Frequencies between Positive and Negative Samples by $Z_{i,j}=Z_{i,j}^{+}-Z_{i,j}^{-}$ Enriched nucleotides correspond to the condition $Z_{i,j}>0$ while depleted to $Z_{i,j}<0$.

First, we plotted enriched and depleted nucleotides of the training datasets in Figure 2, which directly reflected the differences of position-specific nucleotide frequencies between positive and negative samples by $Z_{i,j}=Z_{i,j}^{+}-Z_{i,j}^{-}$ (i.e., the position-specific nucleotide propensity (PSNP) matrix described in Materials and Methods). Obvious differences can be observed between m5C and non-m5C sequences as well as upstream and downstream regions. Generally, the C and U bases are almost enriched in positive samples, whereas the A and G bases are almost enriched in negative sequences. However, nucleotides near the center (C, labeled as 0) show a completely different distribution, where C and U are more likely located in negative samples at positions 1, 2, and 4 and −6, −3, −2 and −1, respectively. At the same time, A and G refer to distribution in positive samples at positions 1, 2, 4, and 10. On the other hand, occupied distinction downstream is obviously weaker than upstream. Specifically, C is, on average, 5% enriched in positive samples, and A is enriched 3% in negative samples upstream. However, the average difference of enriched and depleted nucleotides is approximately 1.4% downstream. It can be generally concluded that the characteristics of nucleotide location between m5C and non-m5C instances can be obviously found; i.e., m5C sites could be identified using the sequence information. Furthermore, the position-specific property is hoped to be an effective feature-extraction method to directly represent RNA sequence.

Many kinds of feature-extraction approaches have been developed to effectively encode RNA sequences, which can be conveniently obtained using several state-of-the-art toolkits, such as Pse-in-One2.0,^34^ BioSeq-Analysis2.0,^35^ iLearn,^36^ PyFeat,^37^ and so forth. Here, four kinds of feature-representing techniques associated with nucleotide frequencies were first investigated, including PSP, Kmer, ENAC, and xxKGap. Corresponding experimental results using the RF classifier are listed in Table 2, where 10-fold CV, and independent tests were used for training (left) and testing datasets (right), respectively. For three kinds of PSP features (i.e., PSNP, PSDP, and PSTP, associated with frequencies of nucleotides, dinucleotides, and trinucleotides, respectively), performances were gradually increased. It can be seen that accuracies over 10-fold CV and independent tests were only 65.48% and 65.05% for PSNP features; however, accuracies of 67.29% and 74.98%, respectively, were quickly achieved for PSTP. Compared to the latest tool, iRNAm5C,^32^ the accuracy over the independent test using only 39-dimensional PSTP features has achieved 74.00%, although it was 3.41% lower over 10-fold CV. Thus, the distribution of trinucleotides is exactly an effective description to represent m5C sequences. As for three Kmer features (i.e., nucleic acid composition [NAC], DNC, and TNC, associated with *k*s = 1, 2, and 3, respectively), predictive accuracies increased with k, where TNC features showed better accuracies of 69.26% and 72.55% for training and testing datasets. As a variation of the NAC technique, ENAC also showed good performances, with accuracies of 69.11% and 71.9% on two datasets. Additionally, xxKGap results were also listed with different conditions, including monoMonoKGap (mMKGap), monoDiKGap (mDGap), and diMonoKGap (dMGap), with *k*s = 1, 2, and 3, corresponding to dinucleotide and trinucleotide frequencies within kgaps. It can be observed that there were not obvious improvements for those listed nine features with k increasing, and mM2Gap showed relatively best performances with 10-fold and independent accuracies of 68.80% and 77.20%.

**Table 2:** Evaluated Performances of Frequency-Associated Feature-Extraction Techniques Using the RF Classifier, Where 10-fold CV, Left, and Independent Tests, Right, Were Separately Used for Training and Testing Datasets

Feature Subset	Training Datasets				Testing Datasets			
	Acc (%)	MCC	Sn (%)	Sp (%)	Acc (%)	MCC	Sn (%)	Sp (%)
PSNP	65.48	0.31	57.78	73.19	65.05	0.32	49.60	80.50
PSDP	65.07	0.31	56.78	73.36	67.52	0.36	57.54	77.50
PSTP	67.29	0.35	61.30	73.28	74.98	0.51	65.87	84.10
NAC	64.96	0.30	61.32	68.60	68.75	0.38	69.70	67.80
DNC	68.74	0.38	64.17	73.30	72.60	0.45	70.40	74.80
TNC	69.26	0.39	61.92	76.59	72.55	0.45	68.90	76.20
ENAC	69.11	0.38	64.53	73.68	71.90	0.44	71.90	71.90
mM1GAP	68.11	0.36	62.94	73.28	71.45	0.43	69.50	73.40
mM2GAP	68.80	0.38	63.32	74.29	77.20	0.55	80.60	73.80
mM3GAP	69.09	0.38	63.75	74.42	73.50	0.47	71.40	75.60
mD1GAP	67.57	0.36	60.33	74.82	72.15	0.44	68.80	75.50
mD2GAP	68.33	0.37	60.92	75.74	72.10	0.44	68.00	76.20
mD3GAP	68.38	0.37	60.41	76.35	72.70	0.46	68.60	76.80
dM1GAP	68.05	0.37	60.52	75.57	72.95	0.46	69.00	76.90
dM2GAP	68.39	0.37	60.37	76.40	72.10	0.44	68.10	76.10
dM3GAP	68.43	0.37	60.35	76.52	73.10	0.46	68.10	78.10

Acc, accuracy.

Additionally, other five kinds of feature vectors, including EIIP, PseEIIP, PC-PseDNC-general (λ=3, ω=0.2), BE, and NCP + ND were also applied for model constructing; the evaluated results are listed in Table 3. It can be found that PseEIIP and PseDNC features performed well among those five approaches, where corresponding training accuracies achieved 69.24% and 68.63% with testing accuracies of 72.60% and 72.65%, respectively. It was also noted that predictive performances of BE were actually unsatisfied, where training accuracy is only 66.55%. For the PC-PseDNC method implemented in Pse-in-One 2.0,^34^ two important parameters, λ and w, were optimized using the grid search (1≤λ≤10 with Δλ=1; 0.1≤w≤1 withΔw=0.1). Combining predictive accuracies and number of features, PC-PseDNC-general (3,0.2) (i.e., λ=3,w=0.2; abbreviated as PC-PseDNC hereinafter) was finally chosen.

**Table 3:** Same as Table 2 but for Other Five Feature-Representing Methods

Feature Subset	Training Datasets				Testing Datasets			
	Acc (%)	MCC	Sn (%)	Sp (%)	Acc (%)	MCC	Sn (%)	Sp (%)
EIIP	66.65	0.34	59.27	74.02	70.85	0.42	68.40	73.30
PseEIIP	69.24	0.39	62.03	76.44	72.60	0.45	68.80	76.40
PC-PseDNC	68.63	0.37	63.47	73.79	72.65	0.45	70.00	75.30
BE	64.37	0.29	57.48	71.26	66.55	0.33	63.60	69.50
NCP + ND	66.67	0.34	60.92	72.41	70.25	0.41	69.30	71.20

Acc, accuracy.

In general, evaluated accuracies were approximately 68%–69% (10-fold CV) and 72%–73% (independent test) for several well-performing features, including PSTP (independent test: 74.98%), DNC, TNC, ENAC, xxKGAP (mM2Gap: independent test, 77.20%), PseEIIP, and PseDNC. It is known that PSP features reflect characteristics of statistical frequencies for positive and negative samples. Thus, the PSP-based model cannot convince researchers if the number of training instances does not reach a certain level. Additionally, compared with the reported tools, evaluated accuracies were not exactly satisfactory. At the same time, a single kind of feature can only indicate one aspect of sequence information. Therefore, we further incorporated multiple kinds of sequence-encoding methods to obtain comprehensive predictors, which can well reflect sequence information of nucleotide frequencies, physiochemical properties, electron-ion interaction, and so forth.

### Predictive Performances Using Combined Features

Based on the discussion earlier, comprehensive predictive performances of multiple features proceeded further and are summarized in Table 4, where the second column “Fea_num” indicates the number of combined features. For the integration of three PSPs “PSNP + PSDP + PSTP,” predictive accuracies were 67.39% and 73.30% over 10-fold CV and independent tests, respectively. Also, 84-dimensional Kmer features “NAC + DNA + TNC” displayed better results (for the 10-fold CV test: accuracy, 69.13%; MCC = 0.39; for the independent test: accuracy, 73.85%, MCC = 0.48). When the two features were integrated as “PSP + Kmer,” training and testing accuracies were rapidly increased to 71.47% and 77.60%, respectively. Besides, when we incorporated all four kinds of frequency-associated features as “PSP + Kmer + ENAC + mM2Gap,” better training and testing accuracies of 71.72% and 78.15%, respectively, were obtained. As for the combination of “PseEIIP + PC-PseDNC,” no better results were obtained. It is also noted that the feature combination of four kinds of feature-extraction methods, “PSP + Kmer + PseEIIP + PC-PseDNC,” showed the best performances (in total, 287 features), where overall accuracies reached 71.77% and 78.30% over 10-fold CV and independent tests, respectively. In addition, ENAC features were also combined with the 287 features mentioned earlier, written as “PSP + Kmer + PseEIIP + PC-PseDNC + ENAC,” where the accuracy of training datasets was only improved 0.59% but −1.55% for testing datasets. If we considered all kinds of features listed in Tables 2 and 3 (for xxKGap, only mM2Gap was included), there were 1,571 features in total, with evaluated accuracies of 71.93% and 75.71% for training and testing datasets, respectively.

**Table 4:** Performances of Combined Features Over 10-fold CV, in Training Datasets, and Independent Tests, in Testing Datasets

Feature Combination	Fea_num^a^	Training Datasets				Testing Datasets			
		Acc (%)	MCC	Sn (%)	Sp (%)	Acc (%)	MCC	Sn (%)	Sp (%)
PSP (PSNP + PSDP + PSTP)	120	67.39	0.35	60.88	73.89	73.30	0.48	63.30	83.30
Kmer (NAC + DNC + TNC)	84	69.13	0.39	63.41	74.85	73.85	0.48	71.80	75.90
PSP + Kmer	204	71.47	0.43	67.01	75.93	77.60	0.56	71.60	83.60
PSP + Kmer + ENAC	352	71.27	0.43	66.50	76.04	76.80	0.54	72.20	81.40
PSP + Kmer + ENAC + MM2Gap	384	71.72	0.44	67.86	75.59	78.15	0.56	74.10	82.20
PseEIIP + PseDNC	83	69.38	0.39	63.26	75.50	72.45	0.45	70.10	74.80
PSP + Kmer + PseEIIP + PseDNC^b^	287	71.77	0.44	67.56	75.99	78.30	0.57	73.90	82.70
PSP + Kmer + PseEIIP + PseDNC + MM2Gap	319	71.73	0.44	67.86	75.60	78.18	0.57	74.10	82.25
PSP + Kmer + PseEIIP + PC-PseDNC + ENAC	435	72.06	0.44	68.05	76.08	76.75	0.54	73.40	80.10
PSP + Kmer + PseEIIP + PC-PseDNC + ENAC + MM2Gap	476	71.74	0.44	67.44	76.04	77.00	0.54	74.50	79.48
All	1,571	71.93	0.44	68.18	75.69	75.71	0.51	74.50	76.92

Acc, accuracy.

a The “Fea_num” column indicates the number of combined features.

b Performances with maximum accuracies.

Considering the number of features and corresponding performances, the integration of four types of features, “PSP + Kmer + PseEIIP + PC-PseDNC,” was finally used to optimize prediction model. Here, four different classifiers, including RF, SVM, AdaBoost, and NB implemented in the scikit-learn package (*sklearn*),^38^ were separately applied to construct predictive models; the results are given in Table 5. It was found that three algorithms—RF, SVM, and AdaBoost—all showed better results, where average accuracies were up to 71.89% and 79.55% for the training and testing datasets. Here, default parameters were used in preliminary experiments, where n_esti = 100 was set as the number of decision trees in the RF method, and C = 1 and gamma = “scale,” (i.e., gamma = 1/(num_fea ⋅ X.var()) were chosen in the SVM method. Among the four listed methods, the SVM classifier gave the overall best performance (10-fold CV: accuracy = 72.72%, MCC = 0.46; independent test: accuracy = 79.90%, MCC = 0.60), where the related AUC values achieved were 0.70 and 0.88, respectively.

**Table 5:** Comparison of Different Classifiers Using the Feature Combination “PSP + Kmer + PseEIIP + PC-PseDNC”

Classifier	Training Datasets					Testing Datasets				
	Acc (%)	MCC	Sn (%)	Sp (%)	AUC	Acc (%)	MCC	Sn (%)	Sp (%)	AUC
RF	71.77	0.44	75.99	67.56	0.79	78.30	0.57	73.90	82.70	0.85
SVM^a^	72.72	0.46	65.46	79.98	0.80	79.90	0.60	79.40	80.40	0.88
AdaBoost	71.19	0.42	68.33	74.04	0.78	80.45	0.61	77.10	83.80	0.88
NB	66.60	0.34	55.08	78.12	0.71	69.82	0.40	73.00	66.63	0.77

Acc, accuracy.

a Performances with maximum accuracies using the SVM algorithm.

### Parameter Optimization and Comparison with Published Predictors

Parameter optimization is also a critical process for improving the performances of constructed models. Here, two important parameters of the SVM method, C and gamma, were simply selected using the dimension-reduction method.^38^ The best performing model was finally obtained with C = 1.5 and default gamma, corresponding to predictive performances (for training datasets: accuracy = 73.06, MCC = 0.47, and AUC = 0.80; for testing datasets: accuracy = 80.15%, MCC = 0.60, and AUC = 0.88).

**Figure 3:**
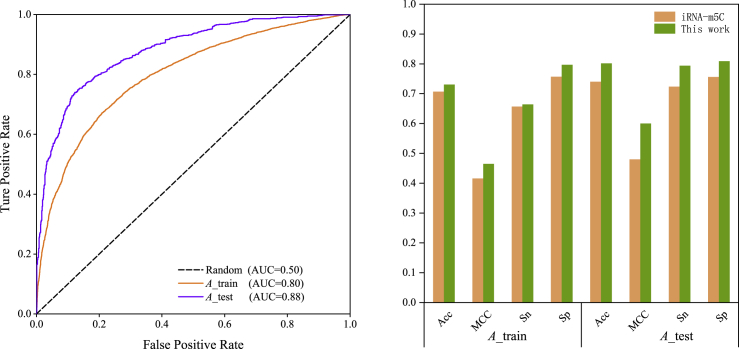
Evaluated Perfromances Left: ROC curves for best performing feature combinations based on the SVM method. Right: comparison of our results (green) and the iRNA-m5C predictor (orange).

Table 6 gave a comparison of our introduced tool iRNA-m5C_SVM and the only two existing predictors, PEA-m5C^31^ and iRNA-m5C,^32^ in *A. thaliana*. For a fair comparison, the same independent datasets in this article were used to obtain performances of the PEA-m5C tool (see details in Lv et al.^32^). It can be seen that only 44.30% accuracy was obtained for the PEA-m5C model.^31^ Compared with the latest iRNA-m5C method,^32^ accuracies were improved from initially 70.70% to finally 73.06% and from 74.0% to 80.15% for training and testing datasets, respectively. Although predictive performances of 10-fold CV only improved 2.36%, the accuracy of the independent test was improved 6.15%. It has been mentioned earlier that the feature combination “KNFC + MNBE + NV” showed the best performance in the iRNA-m5C^32^ predictor. However, besides the basic Kmer technique, the sequence information on PSP, electron-ion interaction potential, and physicochemical properties was considered in this method. At the same time, we also optimized the parameters of the SVM classifier to obtain the best results. Figure 3 visually demonstrated ROC curves of this method (left) and comparison between the latest iRNA-m5C tool^32^ and our method (right). The AUC values for training and testing datasets achieved were 0.80 and 0.88, respectively, where the iRNA-m5C tool^32^ reported AUC values of 0.77 over 10-fold CV. It is believed that our methods can obtain higher accuracies for m5C identification than two existing tools in *A. thaliana*. It is hoped that new benchmark datasets will be collected further with larger amounts of experiment-proved m5C sequences. Then, a more accurate machine-learning-based predictor can be established to predict m5C sites. On the other hand, although, in total, seven kinds of features have been investigated, there are still other powerful feature-extraction techniques worth exploring. Efficient machine learning classifiers and even deep learning methods also should be considered to improve performances.

**Table 6:** Comparison of the Constructed Model with Two Published Methods

Method	Training Datasets					Testing Datasets				
	Acc (%)	MCC	Sn (%)	Sp (%)	AUC	Acc (%)	MCC	Sn (%)	Sp (%)	AUC
PEA-m5C^a^						44.30	−0.11	43.20	45.40	
iRNA-m5C	70.70	0.42	65.70	75.70	0.77	74.00	0.48	72.40	75.60	
This work	73.06	0.47	66.42	79.70	0.80	80.15	0.60	79.40	80.90	0.88

Acc, accuracy.

a Results of the PEA-m5C tool^31^ were excerpted from Lv et al.^32^ (i.e., obtained using independent data objectively).

### Conclusions

As an important post-transcriptional modification, m5C plays crucial roles in the biological process. In this work, multiple sequence features were combined to construct a comprehensive SVM-based model to predict RNA m5C sites in *A. thaliana*. Specifically, four better performing feature-extraction techniques were incorporated, including PSP (PSNP, PSDP, and PSTP), nucleotide composition (NAC, DNC, and TNC), electron-ion interaction pseudopotentials of trinucleotide (PseEIIP), and physicochemical-property-incorporated dinucleotide composition (PC-PseDNC-general). Finally, the optimal model showed a prediction accuracy of 73.06%, with an AUC of 0.80 over 10-fold CV. As for the independent test, the accuracy achieved 80.15%, with an AUC of 0.88. Compared with the latest iRNA-m5C predictor, the evaluated accuracy was improved 4.25% on average. Although there is still some room for further improvement, we believe that the proposed model can be a useful choice to predict m5C sites in RNA sequences.

## Materials and Methods

### Datasets

In this study, benchmark datasets constructed by Lv et al.^32^ were applied, including 6,289 positive and 6,289 negative sequences. Specifically, positive samples were selected from Gene Expression Omnibus (GEO) datasets (https://www.ncbi.nlm.nih.gov/geo/) using the accession number GEO: gse94065,^39^ where the CD-HIT package^40^ was adapted to remove redundant sequences with a threshold of 80%. Then, 6289 negative samples were randomly chosen from their genomes to construct balanced benchmark datasets. Finally, 1,000 positive and 1,000 negative samples were randomly selected as independent datasets, and the rest were treated as training datasets, including 5,289 positive and 5,289 negative sequences (see details in Lv et al.^32^).

### Feature-Extraction Methods

In the process of constructing a machine-learning-based predictor, feature extraction plays an extremely crucial role. In this paper, seven kinds of feature-encoding methods were chosen to represent the sequence information described as follows.

### PSP

PSP is an effective nucleotide-encoding approach that has been successfully applied to the identification of many functional sites in biological sequences.^41^, ^42^, ^43^, ^44^ In this method, the position-specific information is well represented using occurrence frequencies in positive and negative samples. Considering an RNA sequence $R=R_{1}R_{2}R_{3}..R_{2\xi +1}$, the PSNP matrix can be written as a [4×(2ξ+1)]-dimensional vector

(Equation 1) $$Z_{PSNP}=\left[Z_{1}Z_{2}...Z_{2\xi +1}\right]=\left[\begin{array}{cccc} Z_{1,1} & Z_{1,2} & ... & Z_{1,2\xi +1}\\ Z_{2,1} & Z_{2,2} & ... & Z_{2,2\xi +1}\\ Z_{3,1} & Z_{3,2} & ... & Z_{3,2\xi +1}\\ Z_{4,1} & Z_{4,2} & ... & Z_{4,2\xi +1} \end{array}\right],$$

where $Z_{i,j}=Z_{i,j}^{+}-Z_{i,j}^{-}$ gives the difference of frequencies of the *i*th nucleotide at the *j*th position between positive $\left(Z_{i,j}^{+}\right)$ and negative $\left(Z_{i,j}^{-}\right)$ samples. Finally, the (2ξ+1)-length RNA sequence can be encoded as

(Equation 2) $$V_{PSNP}={\left[f_{1}\;f_{2}\ldots f_{j}\ldots \;f_{2\xi +1}\right]}^{T}$$

Here, $f_{j}$ is the element from the $Z_{PSNP}$ matrix

(Equation 3) $$f_{i}=\{\begin{array}{c} Z_{1,j}\;\;\;when\;N_{j}=A,\\ Z_{2,j}\;\;\;when\;N_{j}=C,\\ Z_{3,j}\;\;\;when\;N_{j}=G,\\ Z_{4,j}\;\;\;when\;N_{j}=U, \end{array}j=1,2,...,2\xi +1\text{. }$$

Similarly, PSDP-associated dinucleotides can be written as a [16×(2ξ)]-dimensional vector

(Equation 4) $$Z_{PSDP}=\left[\begin{array}{cccc} Z_{1,1} & Z_{1,2} & ... & Z_{1,2\xi }\\ Z_{2,1} & Z_{2,2} & ... & Z_{2,2\xi }\\ ... & ... & ... & ...\\ Z_{16,1} & Z_{16,2} & ... & Z_{16,2\xi } \end{array}\right]$$

The corresponding feature can be expressed as

(Equation 5) $$V_{PSDP}={\left[f_{1}\;f_{2}\;..\;f_{2\xi }\right]}^{T},$$

and PSTP-associated trinucleotides are displayed as a [64×(2ξ−1)]-dimensional vector,

(Equation 6) $$Z_{PSTP}=\left[\begin{array}{cccc} Z_{1,1} & Z_{1,2} & ... & Z_{1,2\xi -1}\\ Z_{2,1} & Z_{2,2} & ... & Z_{2,2\xi -1}\\ ... & ... & ... & ...\\ Z_{64,1} & Z_{64,2} & ... & Z_{64,2\xi -1} \end{array}\right].$$

The RNA sequence can be represented as

(Equation 7) $$V_{PSTP}={\left[f_{1}\;f_{2}\;..\;f_{2\xi -1}\right]}^{T}.$$

### Kmer

Kmer is a common method to represent RNA sequences, which is simply expressed as the occurrence frequencies of *k*-neighboring nucleotides in bioinformatics.^31^^,^^32^^,^^35^^,^^45^ Here, we considered three kinds of feature vectors with *k*s = 1, 2, and 3, corresponding to NAC, DNC, and TNC, respectively.

### ENAC

The ENAC is a variant of the NAC method, which calculates nucleotide occurrence frequencies in a length-fixed sequence window.^46^ The window can continuously loop through all nucleotides from 5′ to the 3′ terminus. Here, the default length 5 was used, forming a [(2ξ+1−4)×4]-dimensional feature vector.

### xxKGAP

xxKGAP composition is a major method implemented in PyFeat,^37^ which considered kgaps in the nucleotide sub-sequences. Frequencies of these sub-sequences are treated as prediction features. Specifically, for mMKGap features, if kgap = 1, the sequence can be encoded as frequencies of X_X, i.e., 4×1×4=16-dimensional features. If kgap = 2, the sequence can be expressed as 4×2×4=32 features. As for dMKGap, there are, in total, $4^{2}\times n\times$4. The number of features are increased with the n. In this paper, in total, nine kinds of features, including mMKGap, mDGKap, dMKGap with *k*s = 1, 2, and 3, were studied.

### EIIP and PseEIIP

The EIIP approach directly uses EIIP values of 4 nucleotides to represent corresponding nucleotides (expressed as EIIP_A_, EIIP_C_, EIIP_G_, and EIIP_U_), which induces (2ξ+1)-dimensional features.

Additionally, the PseEIIP vector can be written as the mean EIIP value of related trinucleotides:

(Equation 8) $$V=\left[EIIP_{AAA}.f_{AAA},EIIP_{AAc}.f_{AAc},\ldots ,EIIP_{UUU}.f_{UUU}\right],$$

where $f_{XYZ}$ and $EIIP_{XYZ}$ are the normalized frequency and associated EIIP value of the *i*th trinucleotide XYZ by $EIIP_{XYZ}=EIIP_{X}+EIIP_{Y}+EIIP_{Z}$. These two methods showed good results for prediction problems.^43^^,^^47^ It is noted that only EIIP values (A, 0.1260; C, 0.1340; G, 0.0806; and T, 0.1335)^48^ were applied in the iLearn package to represent the DNA sequence.^36^ Here, we still use the EIIP value 0.1335 for the U nucleotide in RNA sequences. It is obviously found that PseEIIP methods produce a 64-dimensional feature vector.

### PC-PseDNC-General

The PC-PseDNC-general method^49^, ^50^, ^51^ incorporates short-range and long-range information by dinucleotide composition and related correlations of physicochemical properties. Here, we extracted PC-PseDNC features by the Pse-in-One 2.0 package with 22 physicochemical properties included,^34^ which can be written as a (16+λ)-dimensional vector

(Equation 9) $$V={\left(x_{1}\cdots \left|x_{16}x_{16+1}\right|\cdots |x_{16+\lambda }\right)}^{T},$$

where the parameter λ indicates the highest counted rank (or tier) in calculations. The detailed description can be found in Liu et al.^34^

### BE

In the BE method, the sequence can be directly written as a 4×(2ξ+1)-dimensional vector, in which A, C, G, and U are characterized as (1, 0, 0, 0), (0, 1, 0, 0), (0, 0, 1, 0), and (0, 0, 0, 1), respectively.^52^, ^53^, ^54^

### NCP + ND

Features NCP and ND are combined to encode RNA sequences with high performances.^55^^,^^56^ The nucleotide N_i_ can be written as

(Equation 10) $$N_{i}=\left(x_{i},y_{i},z_{i},d_{i}\right),$$

where $x_{i}$, $y_{i}$, and $z_{i}$ indicate the three properties of ring structure, functional group, and hydrogen bond, respectively. It is defined as:

(Equation 11) $$x_{i}=\{\begin{array}{c} 1,\;N_{i}\in \left[A,G\right]\\ 0,\;N_{i}\in \left[C,U\right] \end{array};y_{i}=\{\begin{array}{c} 1,\;N_{i}\in \left[A,C\right]\\ 0,\;N_{i}\in \left[G,U\right] \end{array};z_{i}=\{\begin{array}{c} 1,\;N_{i}\in \left[A,U\right]\\ 0,\;N_{i}\in \left[C,G\right] \end{array}\;\;\;\;$$

Additionally, $d_{i}$ is the accumulated density

(Equation 12) $$d_{i}=\frac{1}{\left\|S_{i}\right\|}\sum_{j=1}^{l}f\left(N_{j}\right),|f\left(N_{j}\right)=\{\begin{array}{c} 1\;,\;\left|if\right|\;N_{j}\;\in \left[A,C,G,U\right]\\ 0,\;other|cases\; \end{array},$$

here, $\left\|S_{i|}\right\|$ is the length of the subsequence ended in the relevant nucleotide.

### Classifiers

Many kinds of machine-learning algorithms have been successfully applied in bioinformatics. Here, we used four classifiers implemented in the *sklearn* package^38^^,^^57^ for comparison, including RF, SVM, AdaBoost, and NB.

#### RF

RF is a popular tree-based ensemble estimator, where the overall predictive accuracy is improved by combining a number of decision tree classifiers effectively.^58^ It has been widely applied in fields of bioinformatics research.^30^, ^31^, ^32^^,^^35^^,^^59^, ^60^, ^61^

#### SVM

SVM is an efficient supervised machine-learning algorithm for classification, regression, and outlier detection.^62^, ^63^, ^64^ It has been successfully applied in prediction subjects.^55^^,^^65^, ^66^, ^67^, ^68^, ^69^, ^70^, ^71^, ^72^, ^73^ In this method, the original input vectors are transformed into a higher Hilbert space by kernel function. Here, the radial basis kernel function (RBF) was chosen to seek the best classification hyperplane.

In comparison, AdaBoost and NB were both used in this work. Specifically, the AdaBoost method is used to try to fit a sequence of weak learners (i.e., models that are only slightly better than random guessing, such as small decision trees) on repeatedly modified versions of the data. The predictions from all of them are then combined through a weighted majority vote (or sum) to produce the final prediction.^74^^,^^75^ The NB method is from a set of supervised learning algorithms based on applying Bayes’ theorem with the independent assumption.^76^ Specifically, Gaussian NB algorithm was implemented for the classifier task.

#### CV Test

For a convenient and fair comparison with the newest predictor iRNA-m5C,^32^ 10-fold CV and independent tests were separately used to evaluate constructed models for training and testing datasets. For the *k*-fold CV, benchmark datasets are equally divided into *k* subsets. Then, the k − 1 subsets are used to train the model, and the remaining one is used to test. This process is repeated *k* times until all subsets are used once for testing. The final performance is an average value of all *k* testing experiments.^77^

#### Performance Evaluation

For the two-label classification, four metrics are usually applied to evaluate performances of the proposed model, formulated as follows:^78^, ^79^, ^80^, ^81^, ^82^, ^83^

(Equation 13) $$\{\begin{array}{cc} S_{n}=1-\frac{N_{-}^{+}}{N^{+}} & 0\leq S_{n}\leq 1\\ S_{p}=1-\frac{N_{+}^{-}}{N^{-}} & 0\leq S_{p}\leq 1\\ Acc=1-\frac{N_{-}^{+}+N_{+}^{-}}{N^{+}+N^{-}} & 0\leq A_{CC}\leq 1\\ MCC=\frac{1-\left(\frac{N_{-}^{+}}{N^{+}}+\frac{N_{+}^{-}}{N^{-}}\right)}{\sqrt{\left(1+\frac{N_{+}^{-}-N_{-}^{+}}{N^{+}}\right)\left(1+\frac{N_{-}^{+}-N_{+}^{-}}{N^{-}}\right)}} & -1\leq MCC\leq 1 \end{array}$$

Here, $S_{n}$, $S_{p}$, $S_{p}$, and MCC indicate sensitivity, specificity, accuracy, and Matthew’s correlation coefficient, respectively. N^+^ and N^−^ indicate the number of positive and negative sequences considered, in which incorrectly predicted samples are labeled as $N_{-}^{+}$ and $N_{+}^{-}$, respectively.

In addition, the graph of the ROC^84^^,^^85^ is also widely used to intuitively display the performance. Specifically, vertical and horizontal coordinates are the true positive rate (TPR) and the false positive rate (FPR), respectively. Then, the AUC can be obtained to objectively evaluate performances of the proposed model.

## Author Contributions

L.X. and H.X. proposed the idea and designed the overall research. L.D. performed the experiments and wrote the manuscript. X.L. and H.D. helped to revise the paper. All authors read and approved the final manuscript.

## Conflicts of Interest

The authors declare no competing interests.
